# Acute tear-film disruption in treatment-naive acute anterior uveitis: A retrospective case-control study

**DOI:** 10.1097/MD.0000000000048825

**Published:** 2026-05-22

**Authors:** Zheren Zhang, Chuankai Fang, Bihua Li

**Affiliations:** a Department of Ophthalmology, Tongxiang First People’s Hospital, Tongxiang City, Zhejiang Province, China.

**Keywords:** acute anterior uveitis, inflammation, meibomian-gland dysfunction, NIBUT, tear-film instability

## Abstract

The study aimed to quantify the acute tear-film disruption in treatment-naive, 1st-onset noninfectious acute anterior uveitis (AAU) and identify independent and modifiable determinants. Retrospective case-control study enrolling 180 AAU eyes and 90 age-/sex-matched healthy controls. Same-day noninvasive tear breakup time (NIBUT), tear meniscus height, Schirmer-I and Ocular Surface Disease Index were extracted. Multivariable linear regression with bootstrap correction determined predictors of shorter NIBUT. Mean NIBUT was 4.7 seconds lower in AAU than controls (5.7 ± 1.5 seconds vs 10.4 ± 1.5 seconds; *P* < .001); 66% of AAU eyes versus 2% of controls had NIBUT ≤ 5 seconds. Tear meniscus height (−81 μm), Schirmer-I (−5.3 mm) and Ocular Surface Disease Index (+24 points) all worsened (*P* < .001). Independent reductions in NIBUT: anterior-chamber cells ≥ 2+ (−1.79 seconds), flare ≥ 3+ (−1.23 seconds), concomitant meibomian gland dysfunction (−0.96 seconds) and daily screen time > 8 hours (−0.59 seconds); adjusted *R*^2^ = 0.38, optimism-corrected *R*^2^ = 0.35. In this proof-of-concept study, 1st-onset AAU was associated with measurable tear-film destabilization driven by intraocular inflammatory load. While these findings suggest a potential role for early ocular-surface protection, effect sizes represent upper-bound estimates requiring confirmation in unscreened populations before routine clinical application.

## 1. Introduction

Noninfectious acute anterior uveitis (AAU) is the most frequent form of anterior uveitis and is characterized by an abrupt breakdown of the blood-aqueous barrier that floods the anterior chamber with cells, protein, and fibrin.^[1]^ Although pain, photophobia, and circum-corneal injection dominate the presentation, a subset of patients also report gritty, burning or foreign-body sensations that overlap with those of dry-eye disease.^[2,3]^ Experimental work has shown that key inflammatory mediators – interleukin-1β, tumor necrosis factor-α and matrix metalloproteinase-9 – can reach the ocular surface within 24 hours through the aqueous-tear turnover pathway.^[4,5]^ Once there, they down-regulate goblet-cell mucin expression, accelerate lipid peroxidation and trigger caspase-3-mediated apoptosis of corneal and conjunctival epithelial cells, all of which destabilize the tear film.^[6,7]^

However, once the scene shifts from bench to bedside, the extent to which acute inflammation immediately perturbs the tear film remains fragmentary and inconclusive. Published single-center studies have been small, often included patients already on treatment, and rarely employed age- and sex-matched controls or systematically correlated ocular-surface metrics with inflammatory activity.^[8–10]^ Consequently, it is still unclear whether the gritty, dry or foreign-body sensations reported by patients reflect measurable tear-film dysfunction, how large any defect might be, and which factors – such as inflammatory intensity, meibomian gland status or early therapeutic differences – could be modified to reverse the process.^[11]^ These unresolved questions leave ample room to develop a precision pathway that extends from “suppressing inflammation” to “protecting the ocular surface.”

To close this gap we performed a retrospective, treatment-naïve, case-control study in consecutive adult patients with 1st-onset, noninfectious acute anterior uveitis. Noninvasive tear breakup time (NIBUT), tear meniscus height (TMH), Schirmer-Ⅰ test and the Ocular Surface Disease Index (OSDI) were compared with those of healthy volunteers matched for age and sex. We hypothesized that the intensity of intraocular inflammation and concomitant meibomian gland dysfunction (MGD) would be associated with acute tear-film instability in AAU, representing potentially modifiable correlates.

## 2. Methods

### 2.1. Study design

A retrospective, treatment-naive, case-control study was conducted at our hospital. The study protocol was approved by the Institutional Review Board of Tongxiang First People’s Hospital (approval No. 2026-040) and adhered to the tenets of the Declaration of Helsinki. The institutional review board waived written informed consent because all data were retrospective, preexisting and anonymised; the study nevertheless adhered to the Declaration of Helsinki.

### 2.2. Participants

Consecutive adult patients aged 18 to 65 years presenting with 1st-onset, AAU between January 2022 and December 2023 were identified from electronic medical records. AAU was defined as the presence of ≤ 7 days of symptoms, ≥1 + anterior chamber (AC) cells on Standardization of Uveitis Nomenclature grading, and absence of hypopyon, keratic precipitate clusters, or posterior synechiae. Infectious causes were excluded by focused history, systemic review, and selective laboratory testing (chest imaging, interferon-gamma release assay, syphilis serology, or anterior-chamber polymerase chain reaction for herpes simplex virus/varicella-zoster virus) performed only when clinically indicated.

Exclusion criteria included: corneal opacity within the central 3 mm, active epithelial disease, concurrent ocular surface disorders (except MGD), systemic autoimmune and immune-mediated inflammatory diseases, contact lens wear within 3 months, topical medication use within 1 month, pregnancy or lactation, and unreliable tear function test results. In bilateral cases, the eye with higher AC cell grade was enrolled; in cases of equal grading, the right eye was selected. For retrospective verification, contact lens history and topical ocular medication use were extracted from the “past-history” field of the 1st-visit note and cross-checked against the hospital’s electronic prescribing database. Patients with undocumented status were classified as “unknown” and retained in a sensitivity analysis.

Healthy controls were recruited from asymptomatic volunteers undergoing routine physical examinations. Eligibility required an OSDI score < 10, NIBUT ≥ 10 seconds, TMH ≥ 250 μm, and Schirmer-I test ≥ 10 mm/5 min, with no history of ocular surgery or systemic disease affecting tear metrics. This stringent selection creates a “super-normal” control group optimized for internal validity (minimizing confounding from subclinical dry eye) but potentially inflating between-group effect sizes.

### 2.3. Clinical and ocular surface assessments

Demographic and clinical data – including best-corrected visual acuity, intraocular pressure, Standardization of Uveitis Nomenclature grading scores, smoking status, and daily screen time – were extracted from medical records. MGD was diagnosed using the 2011 Tear Film and Ocular Surface Society Dry Eye Workshop criteria: ≥2 lid-margin abnormalities and expressibility ≥2/8 central glands with turbid or purulent secretion (grade ≥ 2) using a standardized 90 g/mm^2^ meibomian gland evaluator applied for 10 seconds.^[12]^ Inter-rater reliability for MGD grading was assessed in 30 randomly selected eyes by 2 masked examiners; the intraclass correlation coefficient (2-way mixed, absolute agreement) was 0.91 (95% confidence interval [CI] 0.86–0.95), indicating excellent agreement.

Tear-film metrics were extracted from same-day archived records; measurements were carried out under constant room temperature (22°C ± 2) and humidity (45%–55%) by ophthalmologists with ≥5 years of anterior-segment experience.

NIBUT: After a complete voluntary blink, the infrared placido disk of the Keratograph 5M (Oculus) was projected onto the central 6 mm cornea. The built-in software automatically timed the 1st distortion of ≥3 consecutive rings; 5 valid recordings were captured and the median was used. Measurements with eye movement or lid saccade within the 1st 5 seconds were discarded.

TMH: A single horizontal 6 mm scan centered on the pupil was acquired with anterior-segment optical coherence tomography (CASIA-2) 3 seconds after a natural blink. The lower tear meniscus was measured perpendicularly from the corneal surface to the upper border of the meniscus at the central 1 mm zone; 3 scans were averaged.

Schirmer-I test (without anesthesia): A sterile strip (Tianjin Jingming, 5 × 35 mm) was placed at the temporal one-third of the lower fornix for 5 minutes with eyes closed under dim light. Wetting length was read to the nearest 0.5 mm with a back-lit ruler.

OSDI: The validated 12-item Chinese version was self-completed before any procedures; scores were converted to 0 to 100 using the standard formula.

Two trained examiners performed all tests in random order and were masked to participant status. Inter-rater reliability was assessed in 30 randomly selected eyes; intraclass correlation coefficients (2-way mixed, absolute agreement) were 0.95 (95% CI 0.91–0.97) for NIBUT, 0.94 (0.90–0.97) for TMH, and 0.93 (0.89–0.96) for Schirmer-I.

### 2.4. Outcome measures

#### 2.4.1. Primary outcome

NIBUT was analyzed as a continuous variable; a cutoff of ≤ 5 seconds was chosen a priori to define marked tear-film instability, as this threshold has been widely used to identify patients at increased risk of superficial punctate keratitis.

#### 2.4.2. Secondary outcomes

(1) TMH − continuous, with TMH <250 μm classified as reduced;
(2) Schirmer-I test − continuous, with ≤10 mm/5 min regarded as hyposecretion;
(3) OSDI − continuous (0–100 scale), with >12 points indicating symptomatic dry eye;
(4) Proportion of eyes exhibiting NIBUT ≤5 seconds − binary endpoint to estimate the clinical burden of acute instability.

### 2.5. Sample size

Sample size was calculated with Open-Epi v3.01 (Emory University Rollins School of Public Health; 2-tailed α = 0.05, power = 90%, allocation ratio 2:1). Pilot data from 30 eyes collected between November 2021 and February 2022 (not included in this analysis) gave a mean NIBUT of 10.3 ± 1.4 seconds in healthy controls and 6.2 ± 1.7 seconds in treatment-naïve AAU patients. A minimum of 162 AAU eyes were required; allowing 10% missing data we aimed to enroll 180 AAU and 90 control eyes.

### 2.6. Statistical analysis

Statistical analyses were conducted in R 4.3.0 (R Foundation for Statistical Computing). Normality was assessed with Shapiro–Wilk tests; continuous variables are presented as mean ± standard deviation or median (interquartile range), and categorical variables as n (%). Inter-group comparisons used independent *t* tests or Mann–Whitney *U* tests, and χ^2^ or Fisher exact tests. Univariable correlations with NIBUT were evaluated with Pearson or Spearman methods.

Age and sex were forced into the multivariable model regardless of univariable significance to preserve the matching structure. Variables were entered in prespecified blocks: demographics (age and sex), inflammation (AC cells ≥2+ and flare ≥3+), meibomian gland dysfunction (MGD present), and screen time > 8 hours/d. MGD management was not modeled as an intervention in this retrospective cohort. Multicollinearity was excluded if variance inflation factor < 5; residuals were inspected for normality and homoscedasticity. Overfitting was quantified by 1000 bootstrap samples (with replacement), and optimism-corrected *R*^2^ with shrinkage factor is reported. Huber-White robust standard errors were additionally calculated to account for potential heteroscedasticity; quantile regression was not performed given adequate normality of residuals and bootstrap stability. Two-sided *P* < .05 was considered significant.

## 3. Results

### 3.1. Participant characteristics

Between March 2022 and November 2023, 212 treatment-naive patients with 1st-onset AAU were screened; 32 failed eligibility, leaving 180 eyes (180 patients) for analysis. Ninety age- and sex-matched healthy volunteers were enrolled as controls. Among the 180 AAU eyes, 171 (95.0%) had documented absence of contact lens wear and topical ocular medication within the preceding 3 and 1 months, respectively; 9 records (5.0%) were incomplete. Missing data were minimal: 9 participants (5.0%) had incomplete contact lens or medication history; all other variables – including primary outcome (NIBUT) and secondary outcomes (TMH, Schirmer-I, and OSDI) – were complete for all 180 participants. Analysis was complete-case only; no imputation was performed given <5% missingness and MCAR assumption for the single incomplete variable. Sensitivity analyses that either included or excluded these 9 cases yielded an identical between-group NIBUT difference of > 4 seconds (*P* < .001), indicating robustness.

Mean age was 42.6 ± 12.3 years and 97 participants (53.9%) were female. Median symptom duration was 4 (3–6) days. AAU eyes had worse logarithm of the minimum angle of resolution best-corrected visual acuity (0.18 ± 0.21 vs −0.02 ± 0.05, *P* < .001) and higher intraocular inflammatory burden: 112 eyes (62.2%) showed ≥2+ AC cells and 74 (41.1%) fibrin flare ≥3+. MGD was almost 3-fold more prevalent in AAU patients than in controls (61.1% vs 27.8%, *P* < .001). Screen time > 8 hours/day was reported by 33.9% of AAU patients versus 21.1% of controls (*P* = .03). Other demographic variables did not differ between groups (all *P* ≥ .10, Table 1).

**Table 1:** Baseline Characteristics (AAU vs Control).

Characteristic	AAU group (n = 180)	Control group (n = 90)	*P*
Age (yr)	42.6 ± 12.3	42.1 ± 11.9	.72
Sex, female, n (%)	97 (53.9)	48 (53.3)	1.00
Symptom duration (d)	4.5 ± 2.1	–	–
Best-corrected visual acuity, logMAR	0.18 ± 0.21	−0.02 ± 0.05	<.001
Intraocular pressure (mm Hg)	14.2 ± 3.1	14.5 ± 2.8	.39
AC cells ≥ 2+, n (%)	112 (62.2)	0 (0)	<.001
Fibrin flare ≥ 3+, n (%)	74 (37.0)	0 (0)	<.001
MGD present, n (%)	110 (61.1)	28 (27.8)	<.001
Smokers, n (%)	41 (22.8)	18 (20.0)	.52
Screen time > 8 h/d, n (%)	61 (33.9)	19 (21.1)	.03

AAU = noninfectious acute anterior uveitis, AC = anterior chamber, logMAR = logarithm of the minimum angle of resolution, MGD = meibomian gland dysfunction.

### 3.2. Comparison of tear metrics

Primary outcome: NIBUT was markedly shorter in AAU eyes (5.7 ± 1.5 seconds) than in controls (10.4 ± 1.5 seconds), with a mean difference of −4.7 seconds (95 % CI −5.1 to −4.3; *P* < .001); these observed values closely matched the pilot estimates used for sample-size calculation (10.3 ± 1.4 seconds and 6.2 ± 1.7 seconds). Given this stringent control selection, sensitivity analyses using relaxed criteria and population-normative data confirmed the directionality of findings but with attenuated effect sizes (Table S1). Sixty-six percent of AAU eyes recorded NIBUT ≤ 5 seconds compared with only 2% of controls (risk ratio 33.0; 95% CI 8.2–133.4). Within the AAU cohort, higher inflammatory grades further reduced NIBUT: eyes with ≥2+ AC cells averaged 5.3 ± 1.4 seconds versus 6.4 ± 1.5 seconds in those with <2+ cells (*P* < .001), and eyes with flare ≥3+ had lower NIBUT than those with flare <3+ (5.1 ± 1.3 seconds vs 6.1 ± 1.5 seconds, *P* < .001) (Table 2). As prespecified in the protocol, we repeated the primary comparison using adjacent NIBUT cutoffs (≤4, ≤6, and ≤7 seconds); effect direction and significance remained unchanged (Table S2), supporting the robustness of the 5 seconds threshold. Secondary tear metrics: Tear meniscus height was reduced in AAU eyes (224 ± 47 μm vs 305 ± 51 μm; *P* < .001), with 61% recording TMH < 250 μm versus 8% of controls. Schirmer-I values were likewise lower (10.5 ± 3.7 mm vs 15.8 ± 4.0 mm; *P* < .001), and 52 % of AAU eyes met the hyposecretion threshold (≤10 mm/5 min) compared with 14 % of controls. OSDI scores averaged 36.2 ± 17.1 in AAU patients – well above the symptomatic cutoff of 12 – versus 11.8 ± 6.9 in controls (*P* < .001) (Table 2).

**Table 2:** Tear metrics by inflammation grade (mean ± SD).

Parameter	Control (n = 90)	All AAU (n = 180)	AC ≥ 2+ (n = 110)	AC < 2+ (n = 70)	Flare ≥ 3+ (n = 65)	*P* vs control
NIBUT (s)	10.4 ± 1.5	5.7 ± 1.5	5.3 ± 1.4a	6.4 ± 1.5	5.1 ± 1.3	<.001
TMH (μm)	305 ± 51	224 ± 47	218 ± 45	234 ± 48	212 ± 44	<.001
Schirmer-I (mm)	15.8 ± 4.0	10.5 ± 3.7	9.9 ± 3.6	11.6 ± 3.7	9.6 ± 3.5	<.001
OSDI score	11.8 ± 6.9	36.2 ± 17.1	38.7 ± 16.8	32.3 ± 17.2	40.4 ± 16.5	<.001

*P* < .001 for AC ≥ 2 + vs AC < 2 + (unpaired *t* test). Data are mean ± SD.

Mean difference (AAU − Control) for NIBUT: −4.7 seconds (95 % CI −5.1 to −4.3); for TMH: −81 μm (−93 to −69); for Schirmer-I: −5.3 mm (−6.1 to −4.5); for OSDI: +24.4 points (+21.1 to +27.7).

Risk ratio for NIBUT ≤ 5 seconds: marked tear-film instability; TMH < 250 μm: reduced tear volume; Schirmer-I ≤ 10 mm/5 min: hyposecretion; OSDI > 12: symptomatic dry eye. AAU = noninfectious acute anterior uveitis, AC = anterior chamber, NIBUT = noninvasive tear breakup time, OSDI = Ocular Surface Disease Index, SD = standard deviation, TMH = tear meniscus height.

When stratified by MGD, AAU eyes with MGD showed higher rates of NIBUT ≤ 5 seconds, TMH < 250 μm, Schirmer-I ≤ 10 mm and OSDI > 12 than MGD-negative eyes (all *P* < .001) (Table 3).

**Table 3:** Abnormality rates in controls and AAU eyes by MGD status n (%).

Parameter	Control (n = 90)	Flare < 3+ (n = 115)	MGD+ (n = 108)	MGD− (n = 72)	*P* *
NIBUT ≤ 5 s	2 (2)	66 (57)	82 (76)	36 (50)	<.001
TMH ≤ 250 μm	7 (8)	70 (61)	81 (75)	38 (53)	<.001
Schirmer ≤ 10 mm	13 (14)	60 (52)	73 (68)	35 (49)	<.001
OSDI > 12	12 (13)	88 (77)	92 (85)	56 (78)	<.001

AAU = noninfectious acute anterior uveitis, MGD = meibomian gland dysfunction, NIBUT = noninvasive tear breakup time, OSDI = Ocular Surface Disease Index, TMH = tear meniscus height.

* *P* < .001 versus control; *P* < .001 MGD+ versus MGD−.

### 3.3. Univariable associations

AC cell grade showed the strongest correlation with NIBUT (*r* = −0.42, *P* < .001), followed by flare grade (*r* = −0.38) and fibrin presence (*r* = −0.30; all *P* < .001, Table 4). Eyes with MGD had a mean NIBUT 1.1 second shorter than those without (95% CI 0.5–1.7, *P* = .002), and screen time >8 hours reduced NIBUT by 0.9 second (*P* = .011). Age, sex and smoking were not significant (*P* ≥ .24).

**Table 4:** Univariable correlations with NIBUT.

Variable	*r*	*P* value
AC cell grade	−0.42	<.001
Fibrin flare grade	−0.38	<.001
Presence of fibrin	−0.30	<.001
MGD present	−0.29	<.001
Screen time > 8 h/d	−0.21	.011
Age	−0.08	.24
Sex (female)	−0.06	.38
Smoking	−0.07	.33

AC = anterior chamber, MGD = meibomian gland dysfunction, NIBUT = noninvasive tear breakup time.

### 3.4. Independent predictors of tear instability

In multivariable linear regression, AC cells ≥2+, flare ≥3+, MGD, and screen time >8 hours/d remained significant independent predictors after adjustment for age and sex (adjusted *R*^2^ = 0.38, *F* = 19.4, *P* < .001; Table 5). The optimism-corrected *R*^2^ was 0.35 and the shrinkage factor 0.92, indicating minimal overfitting. Full bootstrap-corrected coefficients are listed in Table S3. Each 1-level rise in AC cells (≥2+) shortened NIBUT by 1.79 second (β = −0.31, *P* < .001), whereas flare ≥3+, MGD and prolonged screen exposure reduced NIBUT by 1.23, 0.96,and 0.59 second, respectively, independent of age and sex. No multicollinearity was detected (variance inflation factor < 1.6). Residual diagnostics showed no substantial deviation from normality (Shapiro–Wilk *P* = .31) or heteroscedasticity (Breusch–Pagan *P* = .42). Bootstrap distributions for all coefficients were symmetric with 95% intervals closely approximating model-based CIs (Table S3), supporting model validity. Thus, intraocular inflammatory burden, rather than demographics, is the principal determinant of acute tear-film breakdown in iridocyclitis.

**Table 5:** Multivariate linear regression: predictors of shorter NIBUT.

Predictor	β (95% CI)	Standardized β	*P* value	VIF
Age	−0.03 (−0.08 to 0.02)	−0.08	.21	1.2
Sex (female)	−0.15 (−0.62 to 0.32)	−0.05	.53	1.3
AC cells ≥ 2+	−1.79 (−2.27 to −1.31)	−0.31	<.001	1.5
Fibrin flare ≥ 3+	−1.23 (−1.71 to −0.75)	−0.26	<.001	1.6
MGD present	−0.96 (−1.44 to −0.48)	−0.20	<.001	1.4
Screen time > 8 h/d	−0.59 (−1.09 to −0.09)	−0.11	.021	1.2

Model: adjusted *R*^2^ = 0.38, *F* = 19.4, *P* < .001. Variables with biological plausibility (age, sex, MGD, and screen time > 8 hours/d) were forced into the multivariable model regardless of *P* value. AC = anterior chamber, CI = confidence interval, MGD = meibomian gland dysfunction, NIBUT = noninvasive tear breakup time, VIF = variance inflation factor.

## 4. Discussion

In treatment-naïve, 1st-onset AAU, the tear film failed across all tested parameters within days of symptom onset. The 4.7 seconds shortening of NIBUT is comparable to the deficit reported in moderate meibomian gland dysfunction and is 3-fold the minimal clinically important difference (−1.5 seconds) established for this metric. Together with the 81 μm reduction in tear meniscus height and the 5-point drop in Schirmer-I, the data indicate an immediate, quantitative shift toward tear-film instability. Whether this acute disruption meets the formal definition of dry-eye disease, which requires persistent symptoms, corneal staining, and repeatable findings, cannot be determined from our single-visit design. Nevertheless, the magnitude of change suggests that 1st-onset AAU produces an “acute dry-eye phenotype” that may transiently share the biomechanical consequences of chronic disease, such as increased risk of punctate keratitis.

Inflammation intensity appears to be the principal driver of acute tear-film breakdown: in our cohort, eyes with ≥2+ AC cells already had a mean NIBUT 1.1 second shorter than those with <2+ cells, and every additional cell grade further reduced NIBUT by 1.79 seconds after multivariable adjustment for demographics and clinical factors. This dose–response pattern mirrors experimental findings that interleukin-1β, tumor necrosis factor-α, and fibrinogen reach the ocular surface within hours via aqueous-tear turnover and directly destabilize the lipid layer.^[13,14]^ A comparable gradient was seen for flare: fibrin flare ≥3+ shortened NIBUT independently of cell grade, consistent with in vitro evidence that pathophysiologically relevant fibrinogen concentrations – mirroring protein levels in high-flare states – impair tear-film stability, and with clinical observations reporting meaningful NIBUT differences between eyes with high versus low flare.^[15]^ Together, these clinical-laboratory parallels support the notion that anterior-chamber grading can serve as a real-time surrogate biomarker for impending ocular-surface failure, even though tear cytokine levels were not assayed in the present study.^[16]^

When contextualizing our findings within existing literature, 2 key contrasts emerge. First, most prior studies recruited patients who had already started topical corticosteroids; evidence from both randomized and observational series indicates that corticosteroid therapy lowers aqueous and tear-fluid pro-inflammatory mediators and can partially restore tear stability.^[17,18]^ Consequently, those reports are likely to have underestimated the pure inflammatory effect we observed. Second, work in chronic or juvenile idiopathic arthritis-related uveitis describes only modest reductions in NIBUT despite high symptom scores, implying that long-standing inflammation may generate neuropathic discomfort rather than measurable tear-film thinning.^[19]^ By restricting recruitment to treatment-naïve, 1st-onset eyes, we removed both the steroid “buffer” and the neural noise inherent in chronic disease, so the 4.7-second NIBUT deficit reported here probably represents the best currently available estimate of tear-film shortening produced solely by acute anterior-chamber inflammation.

in vitro interferometry shows that physiologically relevant fibrinogen concentrations (1–4 mg/mL) increase tear evaporation by 15% to 20%, presumably by forming a surface protein lattice that collapses the lipid layer.^[20,21]^ Physiologic tear fibrinogen is an order of magnitude lower, and even flare ≥3+ corresponds to only ~0.2 to 0.5 mg/mL in the anterior chamber.^[22]^ Whether such submilligram levels can amplify evaporative loss remains unknown; however, the consistent 1-second NIBUT difference between high- and low-flare eyes with similar cell counts suggests a biophysical contribution that warrants in vivo lipid-layer interferometry confirmation.^[23]^ If verified, rapid suppression of proteinaceous flare (e.g., intensive topical corticosteroid every 2 hours) would offer a mechanistically rational adjunct to conventional cell-directed therapy.

Meibomian gland dysfunction was present in 61% of AAU eyes versus 28% of controls, and acted as an independent “second hit.” Two explanations, not mutually exclusive, are plausible: inflammatory mediators arriving at the lid margin suppress meibocyte differentiation and promote ductal keratinization, accelerating obstructive MGD^[24]^; a preexisting deficient lipid layer lowers the cytokine threshold at which aqueous tears break up. A recent lipid-layer interferometry study quantified that a 30 nm reduction in lipid thickness shortens NIBUT by 1.2 seconds under identical cytokine exposure – remarkably close to our regression coefficient of −0.96 second for MGD.^[25]^ Regardless of directionality, the data indicate that addressing MGD at 1st presentation – warm compresses, lid-margin debridement or short-course oral doxycycline – could attenuate the inflammatory amplification loop and hasten surface recovery.^[26]^

Daily screen exposure >8 hours was reported by one-third of AAU patients and conferred an extra−0.59 second reduction in NIBUT after adjustment for inflammation and MGD, a decrement that falls below the minimal clinically important difference. Nevertheless, the effect is fully reversible and may accumulate with other interventions^[27]^; counseling patients on the 20-20-20 rule (20-second break every 20 minutes, looking 20 ft away) therefore remains a zero-cost, no-risk adjunct that can be implemented at the slit-lamp without delaying primary antiinflammatory therapy.^[28]^

Taken together, the findings support a dual-hit model in AAU: cellular inflammation delivers proteolytic cytokines,^[5]^ proteinaceous flare increases evaporative load,^[21]^ MGD subtracts the protective lipid layer,^[25]^ and prolonged screen use amplifies oxidative and mechanical stress.^[28]^ All 4 factors are quantifiable within the 1st visit and potentially modifiable. Current guidelines rightly prioritize rapid suppression of intraocular inflammation; our data add the corollary that simultaneous surface protection may prevent chronic postinflammatory dry eye.^[29]^ A prospective cohort pilot found that preservative-free artificial tears q.i.d. plus warm compresses twice daily achieved normal NIBUT within 4 weeks, whereas eyes receiving conventional treatment without heat therapy required a longer time to reach comparable values.^[30]^

A methodological limitation is the use of a “super-normal” control group selected to meet strict thresholds on the same metrics used as study outcomes (NIBUT ≥ 10 seconds, TMH ≥ 250 μm, Schirmer-I ≥ 10 mm, and OSDI < 10). This design choice, while optimizing internal validity by excluding subclinical dry eye, structurally inflates between-group differences, relative risks, and effect sizes compared to unselected population controls. Indeed, mild tear-film instability is prevalent in otherwise healthy adults, particularly with prolonged screen exposure. We addressed this through sensitivity analyses demonstrating that the AAU-control NIBUT difference remained clinically meaningful (2.3–4.7 seconds) across relaxed control definitions and population-normative external data, though effect sizes attenuated by 38% to 51% with less stringent selection. Consequently, these findings demonstrate the biological plausibility of acute tear-film disruption in AAU, but do not provide estimable excess risk applicable to routine clinical settings. Whether observed associations translate into clinically meaningful diagnostic thresholds or therapeutic targets requires prospective validation in unscreened, population-representative cohorts.

These findings establish the 1st quantifiable link between acute intraocular inflammation and immediate tear-film failure, yet several constraints attendant to our design merit explicit mention. This work, by nature of its retrospective design, carries the usual constraints of observer-dependent grading and missing confounders. Tear cytokine sampling was not possible, so the proposed aqueous-to-tear pathway remains indirect and awaits multiplex or micro-dialysis confirmation in prospective cohorts. A single-center Han-Chinese sample and moderate ambient humidity may also limit generalisability to drier regions or other ethnicities. Screen time exposure was self-reported and may not precisely quantify actual exposure; objective device-tracking would strengthen future studies. Dichotomization of inflammatory grades at clinically relevant thresholds sacrifices statistical power; future studies should employ ordinal or spline models to characterize dose–response relationships. Systemic autoimmune disease was excluded at baseline per protocol, and all 180 analyzed patients were free of these conditions at presentation. This study therefore does not address whether acute tear-film instability predicts subsequent systemic disease onset in initially seronegative individuals – a distinct research question requiring prospective longitudinal cohorts with extended follow-up beyond the acute uveitic episode. Longer-term outcomes remain uncharted, and future studies that track tear physiology beyond the acute phase will clarify whether early surface support merely hastens symptom relief or also reduces the later burden of chronic dry-eye.

## 5. Conclusion

Noninfectious acute anterior uveitis was associated with tear-film disruption in this proof-of-concept study, with higher AC cell grade, fibrin flare ≥3+, concomitant MGD and screen time >8 hours as independent correlates. Whether integrating early surface protection with standard antiinflammatory therapy improves outcomes remains hypothetical; prospective randomized trials with unscreened controls are needed to test this.

## Author contributions

**Conceptualization:** Zheren Zhang, Chuankai Fang, Bihua Li.

**Data curation:** Zheren Zhang, Chuankai Fang, Bihua Li.

**Formal analysis:** Chuankai Fang.

**Investigation:** Zheren Zhang, Chuankai Fang, Bihua Li.

**Methodology:** Bihua Li.

**Project administration:** Chuankai Fang.

**Resources:** Zheren Zhang, Bihua Li.

**Supervision:** Zheren Zhang, Bihua Li.

**Visualization:** Chuankai Fang.

**Writing – original draft:** Zheren Zhang, Chuankai Fang, Bihua Li.

**Writing – review & editing:** Bihua Li.






