# Supplementary material for: Acute tear-film disruption in treatment-naive acute anterior uveitis: A retrospective case-control study
Source: Medicine (Baltimore). 2026 May 22;105(21):e48825. doi: 10.1097/MD.0000000000048825 (PMC13200963; doi:10.1097/MD.0000000000048825)
Supplement: Supplementary file 1 [file medi-105-e48825-s001.docx]

**Supplement Table 1. Sensitivity Analysis: Impact of Control Selection on Between-Group Comparisons**

| **Analysis** | **Control Definition** | **n** | **Control NIBUT**  **(s)** | **AAU NIBUT**  **(s)** | **Mean Difference**  **(95% CI)** | **Cohen's d** | **Interpretation** |
| --- | --- | --- | --- | --- | --- | --- | --- |
| A (Original) | All thresholds met: NIBUT≥10s, TMH≥250µm, Schirmer-I≥10mm, OSDI<10 | 90 | 10.4 ± 1.5 | 5.7 ± 1.5 | −4.7 (−5.1 to −4.3) | 3.13 | Super-normal reference; upper-bound effect estimate |
| B (Relaxed) | OSDI<13 (mild symptoms allowed) plus ≥2 other thresholds met; no clinical ocular surface disease diagnosis | 38 | 8.6 ± 2.0 | 5.7 ± 1.5 | −2.9 (−3.6 to −2.2) | 1.64 | Intermediate stringency; effect persists with attenuation |
| C (Population-normative) | Pooled normative estimate from TFOS DEWS II unscreened cohorts | — | 8.0 ± 2.5 | 5.7 ± 1.5 | −2.3 (−2.9 to −1.7) | 1.15 | Real-world reference; conservative effect estimate |

Analysis B represents non-overlapping subjects from the same recruitment pool as Analysis A, excluded due to mild OSDI elevation (10–12 points) or single threshold miss. Analysis C derived from Wolffsohn JS, et al. TFOS DEWS II Diagnostic Methodology report. Ocul Surf. 2017;15(3):539-574 [14]. Cohen's d calculated using pooled standard deviation. The AAU–control NIBUT difference remains clinically meaningful (exceeding the minimal clinically important difference of 1.5 s) across all control definitions. Effect sizes attenuate by 38% with relaxed criteria and 51% with population-normative reference, confirming that original estimates represent upper-bound values.
