# Supplementary material for: Acute tear-film disruption in treatment-naive acute anterior uveitis: A retrospective case-control study
Source: Medicine (Baltimore). 2026 May 22;105(21):e48825. doi: 10.1097/MD.0000000000048825 (PMC13200963; doi:10.1097/MD.0000000000048825)
Supplement: Supplementary file 2 [file medi-105-e48825-s002.docx]

**Supplement Table 2.** Sensitivity analysis of between-group differences using alternative NIBUT cut-offs

| NIBUT cut-off | AAU eyes n/N (%) | Control eyes n/N (%) | Risk ratio (95 % CI) | P |
| --- | --- | --- | --- | --- |
| ≤ 4 s | 52 / 180 (28.9) | 1 / 90 (1.1) | 26.0 (3.7 – 183.9) | <0.001 |
| ≤ 5 s * | 119 / 180 (66.1) | 2 / 90 (2.2) | 29.8 (7.6 – 117.0) | <0.001 |
| ≤ 6 s | 151 / 180 (83.9) | 6 / 90 (6.7) | 12.6 (5.7 – 27.8) | <0.001 |
| ≤ 7 s | 168 / 180 (93.3) | 11 / 90 (12.2) | 7.6 (4.4 – 13.2) | <0.001 |

*Primary endpoint cut-off. NIBUT:Non-invasive tear break-up time; AAU: Non-infectious Acute Anterior Uveitis
