# Supplementary material for: Acute tear-film disruption in treatment-naive acute anterior uveitis: A retrospective case-control study
Source: Medicine (Baltimore). 2026 May 22;105(21):e48825. doi: 10.1097/MD.0000000000048825 (PMC13200963; doi:10.1097/MD.0000000000048825)
Supplement: Supplementary file 3 [file medi-105-e48825-s003.docx]

**Supplement Table 3:** Bootstrap-corrected multivariable coefficients (1 000 resamples)

| Predictor | Original β (95% CI) | Shrinkage factor |
| --- | --- | --- |
| Age | −0.03 (−0.08 to 0.02) | 0.92 |
| Sex (female) | −0.15 (−0.62 to 0.32) | 0.92 |
| AC cells ≥2+ | −1.79 (−2.27 to −1.31) | 0.92 |
| Flare ≥3+ | −1.23 (−1.71 to −0.75) | 0.92 |
| MGD present | −0.96 (−1.44 to −0.48) | 0.92 |
| Screen time >8 h/day | −0.59 (−1.09 to −0.09) | 0.92 |

Optimism-corrected R² = 0.35.

NIBUT:Non-invasive tear break-up time; TMH: tear meniscus height; OSDI:Ocular Surface Disease Index;MGD: meibomian gland dysfunction;AAU: Non-infectious Acute Anterior Uveitis;AC: anterior chamber
